# Factors influencing nurses’ intention to work in the oncology specialty: multi-institutional cross-sectional study

**DOI:** 10.1186/s12904-021-00764-9

**Published:** 2021-05-20

**Authors:** Omar Alrasheedi, Timothy John Schultz, Gillian Harvey

**Affiliations:** 1 Adelaide Nursing School, Faculty of Health and Medical Sciences, The University of Adelaide, Adelaide, South Australia 5000 Australia; 2 College of Nursing, King Saud University, Riyadh, 11451 Saudi Arabia; 3 Flinders Health and Medical Research Institute, Flinders University, Bedford Park, South Australia 5042 Australia; 4 College of Nursing and Health Sciences, Flinders University, Bedford Park, South Australia 5042 Australia

**Keywords:** Saudi Arabia, Oncology nursing, Palliative care nursing, Nursing workforce, Nursing turnover, Nursing shortage

## Abstract

**Background:**

Nursing care for terminally ill cancer patients is routinely provided by oncology nurses in Saudi Arabia. Shortages and retention of oncology nurses is an important concern for healthcare leaders.

**Objectives:**

To identify and describe predictors of nurses’ intention toward working in the oncology specialty amongst three groups: undergraduate nursing students, oncology registered nurses and postgraduate oncology nursing students. In particular, the study sought to analyse association between individual characteristics, job-related factors, palliative care knowledge, attitude toward caring for dying patients, general self-efficacy, job satisfaction and intention to work in oncology.

**Methods:**

A cross-sectional study was conducted involving 477 participants in five major hospitals in Saudi Arabia. The Palliative Care Quiz for Nursing, Frommelt Attitudes Toward Care of the Dying Scale, General Self-Efficacy Scale and Minnesota Satisfaction Questionnaire short form were used for data collection. Multilevel logistic regression analysis was used to identify predictors associated with intention to work in oncology.

**Results:**

43.9% (*n* = 208) of the sample reported an intention to work in oncology. Only one variable was a significant predictor of intention to work in oncology across all three groups studied: a more positive attitude toward caring for dying patients (Odds ratio (OR) = 1.09 [95% confidence interval (CI) 1.04–1.16]), (OR = 1.08 [95% CI 1.04–1.12]), (OR = 1.078 [95% CI 1.053–1.103] with *P* ≤ 0.001 for undergraduate, registered and postgraduate groups respectively. At post-graduate level, higher levels of palliative care knowledge and general self-efficacy were significantly associated with increased intention, whilst at undergraduate level, general self-efficacy was a significant predictor. Job satisfaction was a significant predictor of intention amongst registered nurses.

**Conclusions:**

Attitude toward caring for dying patients and general self-efficacy appear to be the most important predictors of intention to work in the oncology nursing specialty. However, the significance of influencing factors varied between the different groups of nurses studied. Perhaps surprisingly, palliative care knowledge was an influential factor amongst the postgraduate group only. The study results provide important insights for nursing leaders and policymakers in Saudi Arabia to inform the future planning of nursing workforce strategies to address shortages and retention of oncology nurses.

## Background

Saudi hospital environments are complicated due to the predominance of an expatriate workforce and patients’ conservative cultural background [1, 2]. While Saudi citizen nurses represent only 36.5% of the total nursing workforce [3], Arabic is the primary language of Saudi Arabia, and only a small number of its citizens are familiar with English. On the other hand, most expatriate nurses are not familiar with the Arabic language, leading to ineffective communication between patients and nurses [4]. These issues may limit hospitals’ abilities to provide high-quality health services and lead to patient dissatisfaction [5].

Nursing care for terminally ill cancer patients is routinely provided by oncology nurses in Saudi Arabia, as specialist palliative care nursing is in its infancy [6]. As oncology services in rural settings are limited, referrals to metropolitan services may cause delayed diagnosis or treatment, and patients consequently presenting with advanced stage cancer [7]. Further, for cultural reasons, Saudi patients may not disclose their cancer diagnosis or prognosis to their family, which may result in delayed treatment and/or cancer-related risks [7]. Although specialised cancer treatment is available for Saudi patients, for reasons specified above, some patients do not seek medical advice except in the late stages of their cancer when the effectiveness of the treatment is minimised, resulting in a high mortality rate and poor prognosis [7].

Shortages and retention of oncology nurses is an important concern for healthcare leaders in Saudi Arabia. Saudi citizens make up less than 9% of the oncology nursing workforce, significantly lower than the proportion of Saudi nurses in the nursing workforce [6, 8]. Expatriate nurses working in oncology have reported difficulties relating to language and cultural barriers especially while delivering end-of-life (EOL) care [7, 9, 10]. Language barriers contribute to poor understanding of information between nurses, the patient and the patient’s family that negatively impact the patient-nurse relationship [11]. In particular, oncology nursing that encompasses caring for a person with a life-threatening illness, experiencing emotional distress, and receiving complex information about chemotherapy, radiotherapy or palliative treatment, necessitates effective and safe communication with patients and families [12]. These issues may negatively affect the nurses’ work environment and contribute to high nursing turnover. In a previous study, a negative correlation was found between nurses perceived self-efficacy and distress amongst a sample of inpatient oncology nurses in the United States [13]. Furthermore, the relocation of expatriate nurses to their home countries requires additional recruitment and training of oncology nurses, creating a financial burden for the Ministry of Health [1]. For these reasons, the Saudi government is undertaking significant changes in the healthcare system through the ‘Saudi Vision 2030’, a national transformation program that aims to increase the proportion of Saudi citizen nurses [3].

A literature review study found there was a negative attitude toward the oncology specialty among novice nurses in the United States, possibly linked to a lack of proper academic preparation and exposure [14]. In New Zealand, Wilkinson [15] conducted a study of 287 newly graduated nurses and found that the oncology specialty was the least preferred place to work following graduation [15]. Part of the explanation for this was that students elected to work in medical and surgical units because they thought that they could consolidate their basic skills before moving to a speciality area. A study in Saudi Arabia evaluated oncology nurses’ attitudes toward caring for dying patients and palliative care knowledge in one of the main referral hospitals. The authors reported that Saudi nurses demonstrated the most negative attitude toward caring for dying patients and poor knowledge compared to nurses from 19 countries working in the same hospital [6].

In summary, there appear to be several issues that could help explain the low representation of Saudi nurses in the oncology nursing workforce, although these have not been fully explored to date within the Saudi context. Better understanding of the factors that affect recruitment and retention of nurses to work in the oncology specialty is important and could help to inform future strategy in line with the ‘Saudi Vision 2030’ [16]. The current study aims to explore these issues in greater depth by examining what influences nurses’ intention to work in the oncology specialty, where intention is defined as a mental process of planning to either work, stay, or leave the oncology specialty [17]. Specifically, to inform the development of a Saudi oncology nursing workforce that can deliver effective palliative care, this study aimed to address two research questions: 1) What are the intentions of three different groups of nurses in Saudi Arabia toward working in the oncology specialty, and 2) what factors influence nurses’ intention toward working in oncology nursing. For the second research question, we tested for associations between potential predictor variables (knowledge, attitude, self-efficacy, job satisfaction) and intention toward working in oncology nursing.

## Methods

### Study design and aims

A cross-sectional study design was employed to analyse the association between: individual characteristics, job-related factors, palliative care knowledge, attitude toward caring for dying patients, general self-efficacy, job satisfaction and nurses’ intention toward working in oncology.

### Setting and sample

Data were collected using convenience sampling from five main hospitals in Saudi Arabia that include King Fahad Medical City (KFMC), King Saud University Medical City (KSUMC), King Faisal Specialist Hospital and Research Centre Riyadh (KFSHRC-R), Prince Sultan Military Medical City (PSMMC) and King Abdullah Medical City (KAMC). KFMC, KSUMC, KFSHRC-R and PSMMC are located in Riyadh and have bed capacity around 1200, 1500, 1500, 1000 respectively. The KAMC is located in Makkah and has around 1500 bed capacity. The sample comprised three groups: undergraduate nursing students (UNS) who had completed their internship program, postgraduate oncology nursing students (PONS) enrolled in a postgraduate oncology nursing diploma program and oncology registered nurses (ORN) currently working in inpatient oncology settings. All PONS had a bachelor degree in nursing and were full-time students as mandated by the responsible authority for postgraduate nursing education in Saudi Arabia. Based on the information provided by the hospitals included in this study, there were approximately 231 UNS, 398 ORN and 36 PONS meeting the inclusion criteria for participation in the study. For the UNS and ORN, the minimum required sample size was estimated at 140 for each group, based on 14 predictor levels and the requirement for at least 10 observations per level [18].

### Recruitment and data collection

Data were collected from June 31 to August 14, 2019. An advertising flyer was used to recruit participants from the selected hospitals. As explained in the flyer, the questionnaire and participant information sheets were placed on the nursing reception desk with instructions to return completed questionnaires to a co-located secure collection box. Boxes were locked by the researcher and emptied at weekly intervals.

### Measurements

The questionnaire comprised a demographic component and working intentions, and four validated instruments to measure knowledge, attitude, self-efficacy and job satisfaction. The first three of these instruments were used with all three groups, whilst the job satisfaction measure was used with the ORN group only.

### Demographics and intention toward working in oncology

Demographic-related questions based on previous studies were designed to collect information about age, gender, marital status and nationality, as well as job-related information [6, 19]. There was a slight variation in the measurement of intention towards working in oncology between the three participant groups to reflect their different situations. UNS participants were asked to report their future intention towards working in oncology and 13 other nursing specialties to understand their preference for oncology within other specialties [15]. For ORN participants, a single item assessed their intention to staying in oncology nursing in the next 3 years and for PONS participants, a single item assessed their intention to working in oncology after degree completion [16]. All three participants groups used a five-point Likert scale ranging from very unlikely to very likely.

### Knowledge

The Palliative Care Quiz for Nurses (PCQN), developed and validated by Ross, McDonald and McGuinness [20], was used to evaluate palliative care knowledge among both qualified nurses and nursing students [21]. The PCQN consists of 20 dichotomous questions in the form of “true” or “false” or “don’t know the answer” with higher scores (out of 20) indicating better knowledge. The internal consistency of the PCQN in this study was acceptable (Kuder–Richardson 20 = 0.70).

### Attitude

Nurses’ attitudes toward caring for dying patients was measured using the Frommelt Attitudes Toward Care of the Dying Scale (FATCOD) [22]. The FATCOD scale consists of 30 statements, and participants are asked to rate each statement on the range of a 5-point Likert scale from strongly disagree to strongly agree. The FATCOD statements are divided into 15 positive and 15 negative statement with a total score ranging from 30 to 150. A high score overall indicates a positive attitude toward caring for dying patients [22]. The internal consistency of the FATCOD in this study was good (Cronbach’s alpha = 0.81). The content validity for FATCOD is 1.00 [22].

### Self-efficacy

The General self-efficacy scale (GSE) developed and validated by Schwarzer and Jerusalem [23] was used to assess the strength of an individual’s belief in their ability to respond to novel or difficult situations and to deal with any associated barriers. The GSE scale has 10 items with a 4-point choice scale ranging from “1 = not at all true” to “4 = exactly true” [23]. GSE has been validated and used in several studies among undergraduate nursing student [24, 25] and among registered nurses [26–28]. The internal consistency of the GSE in this study was good (Cronbach’s alpha = 0.85).

### Job satisfaction

The Minnesota Satisfaction Questionnaire (MSQ) Short-Form was used to evaluate employees’ feelings toward their job [29, 30]. The MSQ short-form comprises 20 statements and participants are asked to rate their feeling on each statement on range from “Very Satisfied = score 5” to “Very Dissatisfied = score 1. The internal consistency of the MSQ in this study was good (Cronbach’s alpha = 0.92). The construct validity of MSQ has been confirmed through data from various occupational groups at the 0.001 significance level on all scales [30].

### Statistical methods

SPSS (IBM, v 25.0) was used for data analysis. Descriptive statistics (frequency analyses of the categorical variables and means and standard deviations for the continuous variables) were used to first summarise the survey responses. Inferential statistics including chi-square for categorical outcomes and t-tests for continuous outcomes were used to identify variables (*P* <  0.20) for subsequent multivariate analysis. A correlation matrix was used to describe the relationship between study variables.

A multilevel logistic regression model was used to assess the effect of individual characteristics, job-related factors, PCQN, FATCOD, GSE and MSQ (dependent variables) on nurses intention toward working in the oncology speciality (independent variables). Backward elimination method was used to fit the regression model, which excluded variables in stepwise fashion in which *P* > 0.5 [31]. All test assumptions such as linearity, normality, collinearity and homoscedasticity were tested.

## Ethics approval and informed consent to participate

This study complied with the Declaration of Helsinki and was approved by The University of Adelaide, Australia Institutional Review Board (IRB) (no. H-2019-078), KFMC IRB (no. 19-250E), KSUMC IRB (no. E-19-4107), KFSHRC-R IRB (no. 2191205), PSMMC IRB (no. HP-01-R079), KAMC IRB (no. 19–553). Informed consent was obtained from all study participants. Completion and return of the questionnaire by the participants indicated their consent to participate in the study as explained in the participation information sheet and the flyer. Participants were informed that they were free to withdraw from the study at any time and that anonymity and confidentiality would be maintained through not using personal identifiers or reporting potentially identifiable information.

## Results

### Univariate and bivariate analysis

The total sample for this study consisted of 474 out of 665 participants equating to a 71.2% response rate. Results for each group are summarised in Table 1 and will be presented separately.

**Table 1:** Univariate and bivariate analysis of the study variables for the UNS, ORN and PONS N or M ± SD

Variables	Nurses’ Intention Toward Working in Oncology Nursing											
	UNS				ORN				PONS			
	Total *N* = 178	Likely *N* = 51	Unlikely *N* = 127	*P-value*	Total *N* = 263	Likely *N* = 130	Unlikely *N* = 133	*P- value*	Total *N* = 33	Likely *N* = 27	Unlikely *N* = 6	*P-value*
Age	23.6 ± 1.2	23.6 ± 1.63	23.6 ± 0.99	0.88^c^	35.3 ± 7.6	36.5 ± 8.1	34.1 ± 7	0.01^c^	30.7 ± 2.7	30.7 ± 2.7	30.3 ± 2.7	0.74^c^
Gender												
Female	130	44	86		233	112	121		25	21	4	
Male	48	7	41	0.012^a^	30	18	12	0.21^a^	8	6	2	0.56^b^
Marital status												
Single	161	46	114		115	53	58		12	9	2	
Married	17	5	12	0.94^a^	148	74	74	0.83^a^	21	17	4	0.86^b^
Nationality												
Saudi	177	51	126		9	4	5		33	27	6	
Non-Saudi	1	0	1	0.52^b^	254	126	128	0.76^b^	0	0	0	n/a
Received undergraduate palliative care education												
Yes	37	7	30		75	42	33		2	2	0	
No	141	44	97	0.14^a^	188	88	100	0.17^a^	31	25	6	0.49^b^
Received palliative care education after graduate												
Yes	n/a				92	60	32		17	15	2	
No	n/a				171	70	101	< 0.001^a^	16	12	4	0.32^b^
Level of nursing education												
Diploma	n/a				37	23	14		n/a			
Bachelor	n/a				215	101	114		n/a			
Postgraduate	n/a				11	6	5	0.22^a^	n/a			
Years as registered nurse*	n/a				12.1 ± 7.3	13.3 ± 7.8	10.9 ± 6.5	0.007^c^	6.7 ± 4	6.63 ± 4.04	6.79 ± 4.35	0.93^c^
Years as oncology nurse*	n/a				7.6 ± 6	8.4 ± 6.3	6.9 ± 5.7	0.04^c^	1.6 ± 2.7	1.68 ± 2.97	1.29 ± 0.84	0.75^c^
in the current hospital*	n/a				6.3 ± 5.6	6.9 ± 6.1	5.6 ± 5	0.62^c^	n/a			
Years in the current unit*	n/a				4.9 ± 5.2	5.4 ± 5.6	4.4 ± 4.6	0.13^c^	n/a			
Times caring for the terminally ill												
Never	n/a				0(0%)	–	–		n/a			
1–2 times/month	n/a				48	26	22		n/a			
1-2times/week	n/a				84	36	48		n/a			
3–5 times/week	n/a				78	42	36		n/a			
> 5 times/week	n/a				53	26	27	0.47^a^	n/a			
Type of patient												
Paediatric	n/a				74	30	44		n/a			
Adult	n/a				189	100	89	0.07^a^	n/a			
Intention to stay in the current hospital												
Unlikely	n/a				143	37	106		n/a			
Likely	n/a				120	93	27	< 0.001a	n/a			
Research Instruments												
PCQN (0–20)	7.1 ± 2.1	7.3 ± 1.6	7.1 ± 2.2	0.48^c^	9.6 ± 1.9	10.1 ± 2.11	9.2 ± 1.8	0.001^c^	11 ± 2.1	11.3 ± 2.1	9.8 ± 1.8	0.13^c^
FATCOD (30–150)	98.2 ± 8.1	102 ± 7.6	97 ± 7.9	<.001^c^	108.7 ± 11.2	113 ± 11.6	104.5 ± 9.1	< 0.001^c^	100.3 ± 8.1	101 ± 8	95.6 ± 7.8	0.12^c^
GSE (10–40)	31.8 ± 5.1	33.1 ± 4.3	31.3 ± 5.3	0.03^c^	30.8 ± 4.9	32 ± 4.8	29.6 ± 4.6	< 0.001^c^	28.3 ± 3.3	28.7 ± 3.4	26.7 ± 2.8	0.17^c^
MSQ (20–100)	n/a				69.38 ± 11.5	73.2 ± 10.2	65.7 ± 11.5	< 0.001^c^	n/a			

* Years of nursing experience, a = Chi-square, b = Fisher’s exact, c = independent t-test, n/a = not applicable

### UNS Group

**Fig. 1:**
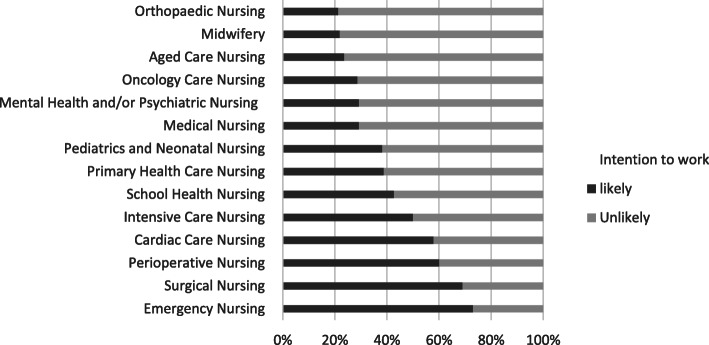
Intention of UNS toward their future nursing career speciality

The UNS consisted of 178 out of 231 participants (77% response rate); the mean age was 23.6(*SD* ± 1.2). The majority (73%, *n* = 130) were female and almost entirely Saudi. Most (79.2%, *n* = 141) participants reported that they did not receive education about palliative care during their undergraduate program. In terms of future intention toward working in oncology and 13 other nursing specialities, emergency nursing, surgical nursing and perioperative nursing were the most preferred nursing specialities among students, whilst oncology nursing, aged care nursing, midwifery and orthopaedic nursing were the least preferred nursing specialities (Fig. 1). Only 51 (28.6%) UNS reported that they were likely (ie a score of 4 or 5 on the Likert scale) to work in oncology nursing. Gender was a significant predictor of likelihood (*P* = 0.012) and females were more likely than males to work in oncology. The FATCOD and GSE scores were significantly higher (*P* <  0.001, *P* = 0.03, respectively) in those who indicated that they were likely to work in oncology (Table 1).

### ORN group

The ORN group consisted of 263 out of 398 participants (66% response rate), with a mean age of 35.3 (SD ± 7.6). The majority of the participants were female (88.6%, *n* = 233) and non-Saudi (96.6%, *n* = 254). Only 28.5% (*n =* 75) of participants reported that they received palliative care education before graduation, and 35% (*n* = 92) reported that they received education after graduation. All participants reported that they delivered nursing care for terminally ill patients at least once per month. The majority of the participants were from adult wards (71.9%, *n* = 189), compared to 28.1% (*n* = 74) from a paediatric ward. About half reported an intention to stay in oncology, and 45.6% (*n* = 120) intended to stay working in their current hospital.

There was a statistically significant association between receiving palliative care education after graduation and intending to stay in the oncology speciality (χ2 = 14.1, *p* <  0.001). The intention of nurses to stay in the current hospital was also significantly associated with intention to stay in the oncology speciality (χ2 = 69.5, *p* <  0.001) (Table 1).

Nurses who were likely to stay in oncology had significantly higher palliative care knowledge (PCQN) (*t* = 3.42, *p* = 0.001), attitudes (FATCOD) (*t* = 6.56, *p* <  0.001), self-efficacy (GSE) (*t* = 4.06, *P* <  0.001) and job satisfaction (MSQ) (*t* = 5.51, *p* <  0.001) than nurses who intended to leave oncology nursing. Nurses who were likely to stay in oncology were older (*t* = 2.54, *p* = 0.012), more experienced as an RN (*t* = 2.71, *p* = 0.007), and more experienced as an ORN (*t* = 2.03, *p* = 0.043).

### PONS group

The PONS group consisted of 33 participants out of 36 participants (91.6% response rate), the mean age was 30.7 (SD ± 2.7). The majority of the participants were female (76%, *n* = 25) and all were Saudi. Most (93.9%, *n* = 31) did not receive education about palliative care during their undergraduate program and most (*n* = 27, 81.8%) intended to work in oncology nursing after graduation (Table 1).

### Pearson’s correlation analysis

#### UNS Group

There was a significant positive relationship between attitude and self-efficacy, indicating that students who had a better attitude toward caring for dying patients also had better self-efficacy (Table 2). The relationship between self-efficacy and palliative care knowledge was also a significant positive relationship, suggesting that students with better palliative care knowledge also had higher self-efficacy.

**Table 2:** Pearson’s Correlation between study variables and outcomes

Variable	AGE	KNOWLEDGE (PCQN)	ATTITUDE (FATCOD)	SELF-EFFICACY (GSE)
AGE	_			
KNOWLEDGE (PCQN)	−0.12	–		
ATTITUDE (FATCOD)	−0.04	0.10	–	
SELF-EFFICACY (GSE)	0.02	0.17^*^	0.15^*^	–

* *P* < 0.05

#### ORN group

There was a significant positive relationship between self-efficacy and: age, years of nursing experience as an RN and as an oncology nurse, years of nursing experience in the current hospital and current unit and job satisfaction (Table 3). Palliative care knowledge was significantly correlated with attitude, age and years of nursing experience as an RN. Job satisfaction was significantly correlated with nursing experience in the current unit, attitude and self-efficacy.

**Table 3:** Pearson’s Correlation between study variables and outcomes for ORNs

Variable	AGE	AS A REGISTERED NURSE#	AS AN ONCOLOGY NURSE#	IN CURRENT HOSPITAL#	IN CURRENT UNIT#	KNOWLEDGE (PCQN)	ATTITUDE (FATCOD)	SELF-EFFICACY (GSE)	JOB SATISFACTION (MSQ)
AGE	–								
AS A REGISTERED NURSE#	.85^**^	–							
AS AN ONCOLOGY NURSE#	.64^**^	.72^**^	–						
IN CURRENT HOSPITAL#	.73^**^	.78^**^	.74^**^	–					
IN CURRENT UNIT#	.59^**^	.66^**^	.66^**^	.81^**^	–				
KNOWLEDGE (PCQN)	.18^**^	.14^*^	.05	.09	.06	–			
ATTITUDE (FATCOD)	.05	.06	−.01	−.08	−.06	.37^**^	–		
SELF-EFFICACY (GSE)	.17^**^	.19^**^	.21^**^	.20^**^	.26^**^	.08	.09	–	
JOB SATISFACTION (MSQ)	.07	.10	.09	.11	.15^*^	.01	.13^*^	.41^**^	–

* *P* < 0.05., ** *P* < 0.01., # YEARS OF NURSING EXPERIENCE

#### PONS group

The only significant relationship of interest was a positive relationship between attitude and age (Table 4).

**Table 4:** Pearson’s Correlation between study variables and outcomes for PONS

VARIABLE	AGE	AS A REGISTERED NURSE#	AS AN ONCOLOGY NURSE#	KNOWLEDGE (PCQN)	ATTITUDE (FATCOD)	SELF-EFFICACY (GSE)
AGE	–					
AS A REGISTERED NURSE#	.74^**^	–				
AS AN ONCOLOGY NURSE#	.21	.58^**^	–			
KNOWLEDGE (PCQN)	−.15	−.16	−.03	–		
ATTITUDE (FATCOD)	.41^*^	.05	−.07	.33	–	
SELF-EFFICACY (GSE)	.18	.003	.038	.018	.182	–

* *P* < 0.05, ** *P* < 0.01, # years of nursing experience

### Multivariate analysis

Only one variable was a significant predictor of intention to work in oncology across all three groups studied: a more positive attitude toward caring for dying patients (Odds ratio (OR) = 1.09 [95% confidence interval (CI) 1.04–1.16]), (OR = 1.08 [95% CI 1.04–1.12]), (OR = 1.078 [95% CI 1.053–1.103] with *P* ≤ 0.001 for UNS,ORN and PONS respectively. Separate multivariate regressions for each participant group follow.

### UNS regression model (intention toward working in oncology)

In multivariate analysis, gender, FATCOD and GSE scores for UNS were significant predictors of intention toward working in oncology nursing (Table 5). Specifically, in terms of gender, the odds of intending to work in oncology nursing were 3.5 times greater for females than they were for males. Regarding FATCOD, every unit increase in the FATCOD score increased the odds of the likelihood to work in oncology by 9%, whilst every unit increase in the GSE score increased the odds of intention toward working in oncology by 6%.

**Table 5:** Multilevel logistic regression examining UNS, ORN and PONS demographic, job-related factors, PCQN, FATCOD, GSE and MSQ on their intention toward working in oncology speciality

Outcome	Predictors		UNS		ORN		PONS	
			Odds Ratio (CI 95%)	*P*- value	Odds ratio (95% CI)	*P-*value	Odds ratio (95% CI)	*P-*value
Intention toward working in oncology speciality.	Age		e		e		e	
	Gender	Male	REF		e		e	
		Female	3.53 (1.84–6.75)	< 0.001	e		e	
	Marital status	Married	e		REF		e	
		Single	e		1.43 (1.25–1.63)	< 0.001	e	
	Nationality	Saudi	n/a		REF		n/a	
		Non-Saudi	n/a		0.336 (0.05–2.43)	0.280	n/a	
	Received undergraduate palliative care education	No	REF		REF		e	
		Yes	3.47 (0.58–20.70)	0.171	1.71 (0.69–4.23)	0.249	e	
	Received palliative care education after graduate	No	n/a		REF		e	
		Yes	n/a		1.56 (0.87–2.77)	0.129	e	
	Type of patient	Paediatric	n/a		REF		n/a	
		Adult	n/a		1.95 (1.60–2.36)	< 0.001	n/a	
	Intention to stay in current hospital	Unlikely	n/a		REF		n/a	
		Likely	n/a		8.49 (3.72–19.34)	< 0.001	n/a	
	Years as registered nurse		n/a		e		e	
	Years as oncology nurse		n/a		1.03 (1.00–1.075)	0.043	e	
	PCQN (0–20)		e		1.10 (0.89–1.37)	0.353	1.272 (1.061–1.525)	0.009
	FATCOD (30–150)		1.09 (1.04–1.16)	0.001	1.08 (1.04–1.12)	< 0.001	1.078 (1.053–1.103)	< 0.001
	GSE (10–40)		1.06 (1.03–1.09)	< 0.001	1.05 (0.99–1.11)	0.102	1.222 (1.044–1.430)	0.013
	MSQ (20–100)		n/a		1.03 (1.02–1.03)	< 0.001	n/a	

n/a = not applicable, e = eliminated as the *p*-value above 0.5

### ORN regression model (intention toward working in oncology)

For ORNs, marital status, type of ward (paediatric versus adult), intention to stay in current hospital, years of experience in oncology, FATCOD and MSQ scores were significant predictors of intention to stay working in oncology (Table 5). Specifically, the odds of staying in oncology were 1.43 times greater in single compared to married nurses. The odds of staying in oncology were 1.95 times greater in nurses working in adult wards compared to paediatrics and 8.5 times greater for staff who reported an intention to stay in the hospital.

Regarding experience in oncology, every year’s increase in the experience of the nurse in oncology increased the odds of the likelihood to stay in oncology by 3%. For the attitude scale (FATCOD), every unit increase in the FATCOD score increased the odds of the likelihood to work in oncology by 8%. Regarding job satisfaction (MSQ), every unit increase in the MSQ score increased the odds of the likelihood to work in oncology by 3%.

### PONS regression model (intention toward working in oncology)

In the PONs sample, the PCQN, FATCOD and GSE scores were significant predictors related to intention toward working in oncology (Table 5). Specifically, every unit increase in the PCQN scores increased the odds of working in oncology by 27%. Every unit increase in the FATCOD score increased the odds of working in oncology by 8%. Regarding self-efficacy (GSE), every unit increase in the GSE scores increased the odds of working in oncology by 22%.

## Discussion

This study examined the factors influencing current and prospective nurses’ intention toward working in the oncology nursing specialty. Overall, intention to work in oncology varied across the three groups studied from a relatively low level in the UNS group to a high level in the PONS, which is not surprising given that they have chosen to undertake a specialist diploma. No previous study has measured the factors influencing nurse’s intention toward working in oncology nursing worldwide. Across all three groups studied, a more positive attitude toward caring for dying patients was a significant indicator of intention to work in oncology. At the post-graduate level, higher levels of palliative care knowledge and general self-efficacy were also significantly associated with increased intention, whilst at undergraduate level, general self-efficacy was a significant predictor of intention. Job satisfaction was a significant predictor of intention amongst the registered nurse sample.

The oncology nursing specialty was one of the least preferred specialty choices among UNS participants, which could be due to factors such as limited student nurse exposure to oncology and their clinical placement experiences [15]. Gender was also significantly related to UNS intention toward working in oncology nursing, with female students reporting that they were more than three times likely to work in oncology than their male counterparts. Studies investigating the relationship between gender and intention to work in oncology are lacking and further research in this area is warranted.

Attitude toward caring for dying patients was the only constant significant predictor across all three groups toward intention to work in oncology nursing. In this study, the FATCOD mean scores for UNS, ORN and PONS were 98.2, 108.7, 100.3, respectively. Table 6 summarises and compares these findings to other studies utilizing the same research instruments in similar populations. Compared to the findings of previous studies, Saudi undergraduate students had slightly higher attitude scores than Turkish students (95.2) [37] and Palestinian students (96.9) [38] but had noticeably lower attitude scores than Greek (111.9) [34], Italian (115) [39] and Swedish students (126) [40]. One explanation for these findings could relate to the influence of religious culture on attitude towards caring for dying patients, given that Muslim countries appear to report lower attitude scores than non-Muslim countries. Again, this is an area where research is lacking and qualitative investigation into the influence of religion on attitude toward caring for dying patients and intention to work in oncology nursing would be beneficial.

**Table 6:** Study variables score in this study and previous studies

Research Instruments	Type of participants	This study	Previous study
PCQN	UNS	7.1 ± 2.1	7.0 ± 2.8 Saudi UNS [32]
			8.0 ± 3.1 Jordan UNS [33]
			8.2 ± 2.8 Greece UNS [34]
	ORN	9.6 ± 1.9	9.1 ± 3.1 Saudi registered nurse [6]
			11.8 ± 2.8 Ireland registered nurse [35].
			11.7 ± 3.1 Australia registered nurse [36]
	PONS	11.0 ± 2.1	n/s
FATCOD	UNS	98.2 ± 8.1	95.2 ± 14.1 Turkish UNS [37]
			96.9 ± 8.3 Palestine UNS [38]
			111.9 ± 10.2 Greece UNS [34]
			115.2 ± 7.86 Italia UNS [39]
			126.0 Sweden UNS [40]
	ORN	108.7 ± 11.2	111.7 ± 14.0 Saudi registered nurse [6]
	PONS	100.3 ± 8.1	n/s
GSE	UNS	31.8 ± 5.1	34.5 ± 8.4 Saudi Arabia UNS [24]
			29.7 ± 4.5 Poland UNS [25]
	ORN	30.8 ± 4.9	29.8 ± 5.8 in Iran registered nurses [26]
			24.9 ± 5.4 in China registered nurses [27]
			24.9 ± 4.4 in China paediatric nurses [28]
	PONS	28.3 ± 3.3	n/s
MSQ	UNS	n/a	n/s
	ORN	69.3 ± 11.5	63.8 ± 15.3Egypt physicians and nurses [41]
			75.0 China psychiatric nurses [42]
	PONS	n/a	n/s

n/a = not applicable, n/s = no previous study

Roleplay simulation in providing EOL care has been shown to have a significant positive impact on nursing and medical students’ attitude [43]. Likewise, a pre-test, post-test study of the effect of EOL simulation found a significant improvement in nursing students’ attitude and perceived competence in the care of dying patients [44]. Furthermore, EOL care simulation was recommended as an educational strategy to improve and evaluate the EOL nursing care competence among students [45]. Although these studies offer encouragement to trial simulation as an intervention to improve attitudes and thereby intention to work in oncology nursing, limitations in their study design, including small sample size, the scope of pilot studies and lack of control groups reduce the strength of such recommendations and further research into EOL simulation is recommended.

Palliative care knowledge was a significant predictor amongst the postgraduate student group only. The PCQN results for UNS and ORN were consistent with the previous studies conducted in Saudi Arabia (Table 6) and relatively low compared with other developed countries such as Australia. This difference could relate to the fact that palliative care education is integrated within undergraduate nursing programs in these other countries [6, 46].

We found that more than half of the ORN participants reported that they were intending to leave their current hospital and/or the oncology specialty within the next 3 years. This is consistent with the results of the job satisfaction scale (MSQ), as the majority were not satisfied with their current nursing job. Moreover, job satisfaction was a significant predictor toward working (staying or leaving) in oncology nursing among ORN participants. Job satisfaction was statistically significantly associated with intention to leave nurses in two studies conducted in China and Jordan [19, 42]. ORN working with adult patients reported an intention to stay working in oncology that was almost double that of paediatric ORNs, possibly due to the additional compassion fatigue associated with caring for children with cancer and their families [47]. The high levels of reported intention to leave oncology nursing amongst the registered nurse sample reinforces the need for urgent planning to decrease nursing turnover and job dissatisfaction among oncology nurses in Saudi Arabia. In subsequent phases of the research, the findings will be discussed in focus group meetings with nursing leaders and educators in Saudi Arabia to identify potential strategies to address the barriers identified in this study and improve the recruitment and retention of Saudi nurses to work in oncology.

### Strengths and limitations

A major limitation is that the use of a cross-sectional study design does not establish a causal relationship between dependent and independent variables. The use of a self-administered questionnaire may have imposed recall bias and social desirability bias [48], however, the low levels of reported intention toward working in oncology nursing would suggest this was not the case. The strength of the study lies in the large sample size and the high response rate of over 70% to the questionnaire survey. The findings add to the body of knowledge on Saudi oncology nursing and can help to inform future recruitment and retention strategies.

## Conclusion

To our knowledge, this is the first study that has investigated the influence of individual characteristics, job-related factors, palliative care knowledge, attitude toward caring for dying patients, self-efficacy and job satisfaction on nurses’ intention toward working in oncology nursing. This study provides a new insight into understanding the oncology nursing workforce in Saudi, in terms of challenges and possible solutions. Findings such as the lack of appeal of the oncology specialty to undergraduate nurses, the likely loss of existing oncology nurses and the importance of attitude in shaping intention to work in oncology nursing, should be taken into account when planning for the future Saudi oncology nursing workforce.

## Data Availability

All datasets during and/or analysed during this study are available from the corresponding author on reasonable request.

## Abbreviations

UNS: Undergraduate Nursing Student
PONS: Postgraduate Oncology Nursing Student
ORN: Oncology Registered Nurse
PCQN: Palliative Care Quiz for Nurses
FATCOD: Frommelt Attitude Toward Care of the Dying Scale
GSE: General self-efficacy scale
MSQ: Minnesota Satisfaction Questionnaire
PCN: Palliative Care Nursing
EOL: End of life
KFMC: King Fahad Medical City
KSUMC: King Saud University Medical City
KFSHRC-R: King Faisal Specialist Hospital and Research Centre Riyadh
PSMMC: Prince Sultan Military Medical City
KAMC: King Abdullah Medical City
